# Variations in Elite Female Soccer Players' Sleep, and Associations With Perceived Fatigue and Soccer Games

**DOI:** 10.3389/fspor.2021.694537

**Published:** 2021-08-25

**Authors:** Frode Moen, Maja Olsen, Gunvor Halmøy, Maria Hrozanova

**Affiliations:** ^1^Department of Education and Lifelong Learning, Faculty of Social and Educational Sciences, Norwegian University of Science and Technology, Trondheim, Norway; ^2^Centre for Elite Sports Research, Department of Neuromedicine and Movement Science, Faculty of Medicine and Health Science, Norwegian University of Science and Technology, Trondheim, Norway; ^3^The Norwegian Football Association, Oslo, Norway

**Keywords:** sleep, soccer, freshness, games, fatigue

## Abstract

The current study investigated the associations between female perceived fatigue of elite soccer players and their sleep, and the associations between the sleep of players and soccer games. The sample included 29 female elite soccer players from the Norwegian national soccer team with a mean age of ~26 years. Perceived fatigue and sleep were monitored over a period of 124 consecutive days. In this period, 12.8 ± 3.9 soccer games per player took place. Sleep was monitored with an unobtrusive impulse radio ultra-wideband Doppler radar (Somnofy). Perceived fatigue was based on a self-report mobile phone application that detected daily experienced fatigue. Multilevel analyses of day-to-day associations showed that, first, increased perceived fatigue was associated with increased time in bed (3.6 ± 1.8 min, *p* = 0.037) and deep sleep (1.2 ± 0.6 min, *p* = 0.007). Increased rapid eye movement (REM) sleep was associated with subsequently decreased perceived fatigue (−0.21 ± 0.08 arbitrary units [AU], *p* = 0.008), and increased respiration rate in non-REM sleep was associated with subsequently increased fatigue (0.27 ± 0.09 AU, *p* = 0.002). Second, game night was associated with reduced time in bed (−1.0 h ± 8.4 min, *p* = <0.001), total sleep time (−55.2 ± 6.6 min, *p* = <0.001), time in sleep stages (light: −27.0 ± 5.4 min, *p* = <0.001; deep: −3.6 ± 1.2 min, *p* = 0.001; REM: −21.0 ± 3.0 min, *p* = <0.001), longer sleep-onset latency (3.0 ± 1.2 min, *p* = 0.013), and increased respiration rate in non-REM sleep (0.32 ± 0.08 respirations per min, *p* = <0.001), compared to the night before the game. The present findings show that deep and REM sleep and respiration rate in non-REM sleep are the key indicators of perceived fatigue in female elite soccer players. Moreover, sleep is disrupted during game night, likely due to the high physical and mental loads experienced during soccer games. Sleep normalizes during the first and second night after soccer games, likely preventing further negative performance-related consequences.

## Introduction

Playing soccer games and high-intensity training sessions at the elite level involves demanding physical efforts with high-intensity sprinting, sudden changes in running speed, accelerations, movement directions, hard tackles, and often maximal jumps (Nédélec et al., 2012). Indeed, both subjectively and objectively quantified physical efforts peak significantly during soccer games (Whitworth-Turner et al., 2018; Lathlean et al., 2019). Such physical efforts lead to muscle damage, which is associated with a marked inflammatory response and upregulated oxidative stress during recovery (Nédélec et al., 2013). This leads, in turn, to the experience of muscular fatigue that reduces maximal voluntary muscle force of players (Gandevia, 2001; Mohr et al., 2005; Reilly et al., 2008), which may lead to a feeling of not being optimally physically recovered.

Soccer games also pose high mental loads, due to demands on concentration and attention, constant awareness of the shifting contextual environment (i.e., the movements of teammates and opponents), and the rapid decision making that is favorable in the play–counterplay interaction against the opponent team (Jordet et al., 2020). When prolonged internal or external demands exceed the capabilities of soccer players to meet these demands (e.g., high amounts of training loads or adversities over time), their respiratory ventilation might increase (Tipton et al., 2017). In addition, high training loads lead to an increase in oxygen uptake during the recovery period after exercise (Børsheim and Bahr, 2003). Respiration rate may also be seen as a physiological correlate of psychological well-being and autonomic reactivity (Sakakibara and Hayano, 1996; Van Diest et al., 2006). Therefore, optimal functioning and recovery are thought to be associated with respiration rate (RP) (Davies and Maconochie, 2009; Nijman et al., 2012; Bennett et al., 2015).

Optimal recovery from mental and muscular fatigue of soccer players contributes to the maintenance and further development of performances in soccer (Bird, 2013; Smith et al., 2018). Sleep is crucial for athletic recovery due to its adaptive and consolidating effects on virtually all systems required for athletic progression (Venter, 2012; Nédélec et al., 2015a). Despite the importance of sleep to athletic performances, soccer players do not necessarily obtain adequate quality and quantity of sleep, especially the night following a soccer game (Fullagar et al., 2015, 2016).

The arousal induced by soccer games is associated with an increased cognitive and emotional activity, which may potentially affect subsequent sleep (Nédélec et al., 2015b). Among elite athletes, competitive periods were associated with poorer sleep quality and more sleep disturbances than non-competitive periods (Biggins et al., 2020). Sleep quantity and quality were shown to be reduced after soccer games (Fullagar et al., 2016; Roberts et al., 2019). After evening soccer games, players obtained less sleep, sleep onset was delayed, and time in bed was shortened (Sargent and Roach, 2016; Lastella et al., 2018; Nédélec et al., 2019). In another study, soccer games influenced sleep more than the start times of games (Lalor et al., 2018). On game night and one night after the game, sleep durations of youth male soccer players were reduced, compared to training days preceding the games (Whitworth-Turner et al., 2018). Further, increases in high-speed running distances during soccer games were associated with increases in sleep durations (+9 min). However, sleep did not vary as a function of game days (Whitworth-Turner et al., 2018).

None of the existing studies investigated how soccer games influence the different sleep stages, comprising light, deep, and rapid eye movement (REM) sleep. This is concerning, since each sleep stage fulfills a distinct yet complementary role in the overall recovery process (Vyazovskiy and Delogu, 2014). Similarly, there is a scarcity of data on female elite soccer players. One recent study among elite female soccer players showed that sleep durations on training days equaled 6 h and 36 min. Following evening soccer games, the sleep durations of the female soccer players were 65 min shorter (Carriço et al., 2018). Thus, the total sleep time was below the recommended threshold for optimal sleep duration (7–9 h in healthy young adults: Hirshkowitz et al., 2015), likely due to interruptions associated with evening soccer games. Beyond these data, the associations between sleep and its stages, and physical and psychological loads in elite female soccer players remain unexplored and vague (Nédélec et al., 2015b).

Building on the gaps in previous research, the current study had two aims. First, we aimed to investigate the associations between perceived fatigue and variations in sleep among female elite soccer players over one full competitive season. Second, we aimed to investigate the influence of soccer games on the sleep of the players. It was expected that perceived fatigue would be associated with disruptions in the sleep of the players and increases in respiration rate (H1). Since soccer games represent the highest load for a female soccer player, it was expected that sleep on the game night would be disrupted, that respiration rate would increase, and that sleep of the players would normalize the nights following game night (H2).

## Method

### Participants

Participants were recruited from the Norwegian female national soccer team. A total of 29 team members were invited to an information meeting about the research project, in which the importance, scope, and the data collection process were explained in detail. Soccer players who showed interest were instructed to sign up for the study and were given a consent form, approved by the local Regional Committee for Medical and Health Research Ethics (REC) board, and an agreement form for the use of equipment. The soccer players who signed and returned these qualified for participation in the study. Twenty-nine soccer players from the Norwegian national team returned the signed consent forms and were enrolled in the study. All 29 players (mean age 25.9 ± SD 4.1, range 20–39 years) completed the study.

### Ethics Statement

All participating athletes gave their informed consent to participate in the study. REC Central, the REC in Central Norway, founded on the Norwegian law on research ethics and medical research, approved the study (project ID 2017/2072/REK midt).

### Instruments

#### Perceived Fatigue

A daily self-report scale based on recommendations for monitoring of overtraining in athletes was used to detect daily experienced fatigue based on their workload that day (Hooper et al., 1995). The use of subjective self-report measures for the measurement of acute, training-related changes in physiological states of athletes over longer periods has previously been recommended (Saw et al., 2016). Players were asked to provide daily scores with visual rating scales on a mobile phone application based on the question “How fatigued do you feel today?” The application used smileys to document the daily scores and visual Likert scales. This method of assessment was previously found to be appropriate and well-accepted by participants (Toepoel et al., 2019). Values ranged from 1 to 10, where “1” indicated low levels of perceived fatigue and “10” indicated extreme levels of perceived fatigue.

#### Sleep

The Somnofy sleep monitor (version 0.7, VitalThings AS, Norway) is a novel, fully unobtrusive tool for sleep assessment, utilizing an impulse radio ultra-wideband (IR-UWB) pulse radar and Doppler technology. Somnofy is certified according to the Federal Communication Commission (FCC) and “Conformité Européene” (CE). The IR-UWB radar emits radio wave pulses in the electromagnetic spectrum, which are able to pass through soft materials (e.g., clothes or duvets), but are reflected by denser materials (e.g., human body). As the pulses are reflected, they are returned and received by the IR-UWB radar again. Then, time-of-flight is used to analyze the time it takes to cover the distance between the radar to the object and then back to the radar. The movement of the sleeping person and their respiration rate are derived from the IR-UWB radar by utilizing the Doppler effect and fast Fourier transform, allowing the Somnofy to monitor the movement and respiration of the individual in bed with high precision. The raw movement and respiration data are processed by a sleep algorithm, which uses machine learning to calculate the relevant sleep variables. Recently, a full validation of Somnofy against manually scored PSG has shown Somnofy to be an adequate measure of sleep and wake, as well as sleep stages, in a healthy adult population (Toften et al., 2020). Sleep studies in athletes, investigating the associations between sleep variables, sleep stages, and physical and mental loads, have shown the Somnofy to be appropriate for use in athletic populations (Hrozanova et al., 2018, 2020; Moen et al., 2020).

For the purposes of this study, the following sleep variables were obtained from the Somnofy sleep monitor: time in bed (TIB), sleep-onset latency (SOL), total sleep time (TST), time in sleep stages (light, LS; deep/slow wave sleep, SWS; and REM), sleep efficiency (SE), and respiration rate during NREM sleep (NREM RPM). A description of these sleep variables is shown in Table 1.

**Table 1:** Complete list of sleep variables derived from the sleep algorithm used in the sleep monitor.

**Sleep variable**	**Abbreviation**	**Units**	**Characteristics of sleep variable**
Time in bed	TIB	h	Total time spent in bed during the night
Sleep-onset latency	SOL	h	The time it takes from when the athlete intends to go to sleep and actually starts to sleep
Total sleep time	TST	h	Total sleep time obtained from sleep onset to time at wake-up
Light sleep	LS	h	Total amount of time in light sleep
Deep sleep	SWS	h	Total amount of time in deep sleep
REM sleep	REM	h	Total amount of time in REM sleep
Sleep efficiency	SE	%	The percentage of total sleep time to lights off and leaving bed
Respiration rate	NREM RPM	N	The number of respiratory ventilations during NREM sleep per minute

### Procedure

Once all participating soccer players returned the signed consent forms, the necessary equipment for sleep monitoring was delivered, along with instructions for correct use. Soccer players were instructed on the correct placement of the sleep monitor and the importance of correct settings for optimal functionality. Data collection lasted for 124 consecutive days and entailed day-to-day monitoring of the sleep of the soccer players and perceived fatigue. Researchers had access to real-time overview of' compliance of participants with the study and monitored the progress closely throughout the whole 124-day period to address and solve any technical issues in relation to the sleep monitoring systems.

Since 29 players participated over 124 days, a total of 3,596 data points with both sleep and fatigue, respectively, could be collected. For objective sleep data, 2,679, or 74.5%, of the potential 3,596 nights of sleep were collected and analyzed. Data were lost due to occasional disengagement of the players with the sleep monitoring systems, technical issues with connecting the Somnofy units to wifi (especially at some hotels), the loss of electricity caused by thunder and lightning, and travels where athletes forgot their sleep monitoring devices at home. Of the 3,596 potential perceived fatigue data, 2,148, or 59.7%, were collected. Data were lost due to the occasional forgetfulness of athletes, and due to the daily demands that reporting their perceived fatigue placed on the athletes. The sleep and fatigue data points, collected over 124 consecutive days, were used to analyze the associations between sleep and perceived fatigue (H1).

Overall, a total of 402 soccer games were played in the investigated period of 124 consecutive days. Each player played 12.8 (SD 3.9) games. The number of played games varied between players, as some players played more games than others (min = 6, max = 19). The number of game nights (G) for all participants equaled 370. Of these, 252 nights of sleep data, or 68.1% of all potential game nights, were collected. The number of nights before the game (G-1) for all participants equaled 362. This is different from G because some games were played on two consecutive days. However, no player played two games on two consecutive days. Of the G-1 sleep data points, 285 nights, or 78.7% of all potential G-1 nights, were collected. The number of first nights after the game (G+1) for all participants equaled 362. Of these, 264 nights of G+1 sleep data, or 72.9% of all potential G+1 nights, were collected. Lastly, the number of second nights after the game (G+2) for all participants equaled 325, again because certain games were not followed by a 2-day break. Of these, 254 G+2 nights, or 78.2% of all potential G+2 nights, were collected. The sleep data collected during G-1, G, G+1, and G+2 were used to analyze the associations between soccer games and sleep (H2).

### Statistical Analyses

Initially, IBM SPSS (version 27.0) was used to conduct demographic and descriptive statistical analyses, which are presented as mean ± standard deviation (SD). Extreme outliers of sleep data, defined as data points >3 box lengths from either hinge of the boxplot, were removed following the visual inspection of boxplots in SPSS. Then, multilevel modeling was used to investigate the associations between perceived fatigue, sleep, and game nights. The collected data created a clustered data structure, in which repeated measurements were clustered within the individual athletes, thus creating dependencies, which, if not taken into consideration in the statistical approach, may cause excessive type I errors and biased parameter estimates. To correct for these risks, multilevel modeling in Mplus, version 8.4 (Muthén and Muthén, 2017), was utilized to carry out the statistical analyses, by clustering the repeated measurements (level 1: sleep and experienced fatigue measurements, game nights) within the athletes (level 2).

Random intercept models were used to investigate the bidirectional associations between sleep and perceived fatigue, in the entire period of the data collection (124 consecutive days). Random intercept models assume that the only variation between individuals is at their intercept and that the effects of the predictor variables do not vary across individuals (fixed slope). To investigate the bidirectional associations between sleep and perceived fatigue, two sets of random intercept models were tested: (1) effects of perceived fatigue (continuous predictor) on sleep (outcome) and (2) effects of sleep (continuous predictor) on the next-day perceived fatigue (outcome). All continuous predictor variables were grand-mean-centered to reduce multicollinearity and to establish a meaningful zero point: The intercept became an average across the whole sample. Further, three sets of random intercept models were used to investigate the variations in sleep across the different game nights: (1) effects of game night (binary predictor: 0 = G-1, 1 = G) on sleep (outcome), (2) effects of first night after the game (binary predictor: 0 = G, 1 = G+1) on sleep (outcome), and (3) effects of second night after the game (binary predictor: 0 = G+1, 1 = G+2) on sleep (outcome).

The results of the random intercept models are presented on the within-athlete and between-athlete level. Associations on the within level refer to the effects of the daily variation within each athlete with the between-level effects (i.e., the average differences between athletes) removed. These results are presented by reporting the estimated effect ± standard error (SE), the associated upper and lower 95% confidence intervals (95% CI [lower value, higher value]), as well as *p*-values. On the between level, the results show the estimated variances of the predictor variables across athletes (i.e., interindividual variances). For each random intercept model, the intraclass correlation (ICC), or the extent to which the dependent values of occasions of measurement in the same participant resemble each other compared to those from different athletes, was calculated. For all random intercept models, *R*^2^ values stating the explained variance on the within level were reported. The alpha level was set at *p* < 0.05 for all random intercept models.

## Results

### Descriptive Statistics of the Entire Period and the Game Periods

**Figure 1:**
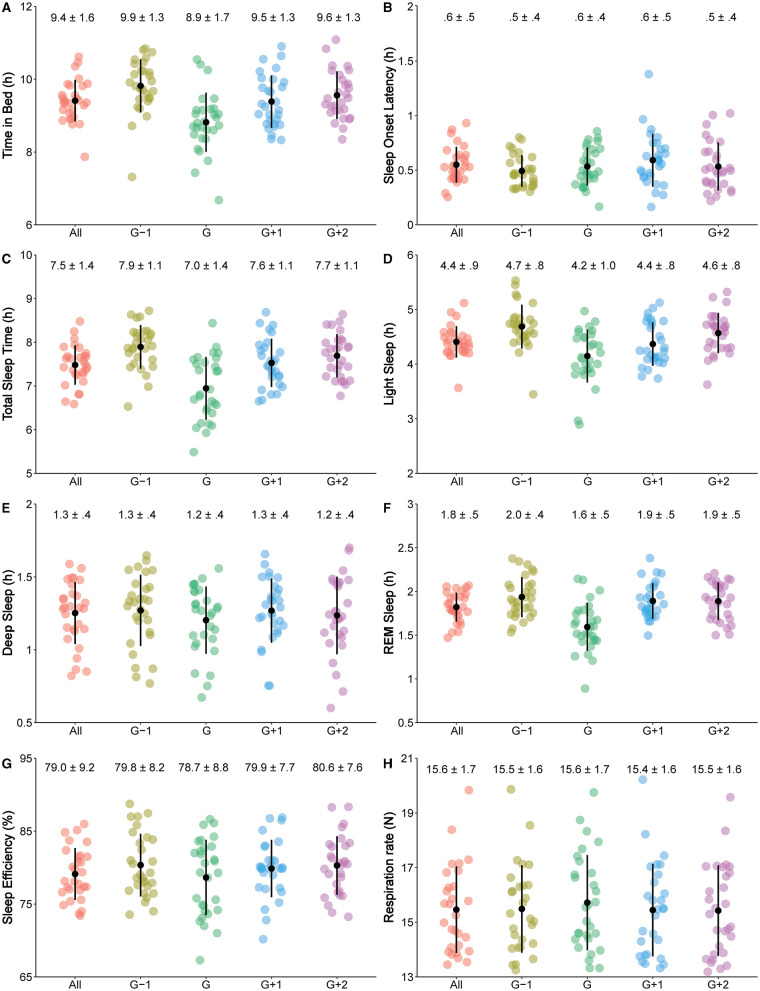
The spread (each participant is represented by a dot), means ± SD (shown by black dot and black line, and numerically given in the upper part of each plot) of the studied sleep variables, which included time in bed **(A)**, sleep-onset latency **(B)**, total sleep time **(C)**, light sleep **(D)**, deep sleep **(E)**, rapid eye movement (REM) sleep **(F)**, sleep efficiency **(G)**, and respiration rate in non-REM sleep **(H)**, during the entire period of data collection (All) and during the game periods (G-1 = night before the game, G = game night, G+1 = first night after the game, G+2 = second night after the game).

Descriptive statistics (mean ± SD, and spread of values) of the studied sleep variables during the entire period of data collection, and during the game periods, are shown in Figure 1. The game periods included the night before the game (G-1), game night (G), first night after the game (G+1), and second night after the game (G+2).

### Bidirectional Associations Between Perceived Fatigue and Sleep

Random intercept models investigating the associations between perceived fatigue (IV) and sleep variables (DVs) showed that with each point increase in perceived fatigue, TIB of athletes increased by 3.6 ± 1.8 min (95% CI [0.07, 7.13], *p* = 0.037). The explained variance of this model was low and equaled 0.4%. The ICC value showed that 7% of the total variance in TIB was due to differences between participants. Further, with each point increase in perceived fatigue, SWS of athletes increased by 1.2 ± 0.6 min (95% CI [0.02, 2.38], *p* = 0.007). The explained variance of this model was low and equaled 0.5%. The ICC value showed that 27% of the total variance in SWS was due to differences between participants. Between-athlete variances were significant for all sleep variables (TIB: *p* = 0.001, SOL: *p* = <0.001, TST: *p* = 0.003, LS: *p* = 0.032, SWS: *p* = <0.001, REM: *p* = <0.001, SE: *p* = <0.001, NREM RPM = <0.001). Perceived fatigue was not associated with variations in any of the other explored sleep variables (see Table A1 for full reports of these results).

Random intercept models investigating the associations between sleep variables (IVs) and the next-day perceived fatigue (DV) showed that with each hour increase in REM, subsequent perceived fatigue decreased by 0.21 ± 0.08 AU (95% CI [−0.37, −0.05], *p* = 0.008). The explained variance of this model was low and equaled 0.5%. The ICC value showed that 28% of the total variance in the next-day perceived fatigue was due to differences between participants. Further, with an increase of one NREM respiration per minute, subsequent perceived fatigue increased by 0.27 ± 0.09 AU (95% CI [0.09, 0.45], *p* = 0.002). The explained variance of this model equaled 7.2%. The ICC value showed that 27% of the total variance in the next-day perceived fatigue was due to differences between participants. Between-athlete variances were significant for all sleep variables (TIB: *p* = 0.002, SOL: *p* = 0.001, TST: *p* = 0.002, LS: *p* = 0.002, SWS: *p* = 0.002, REM: *p* = 0.002, SE: *p* = 0.002, NREM RPM = <0.001). None of the other sleep variables were associated with variations in the next-day perceived fatigue (see Table A2 for full reports of these results).

### Variations in Sleep Across Competitive Periods

**Figure 2:**
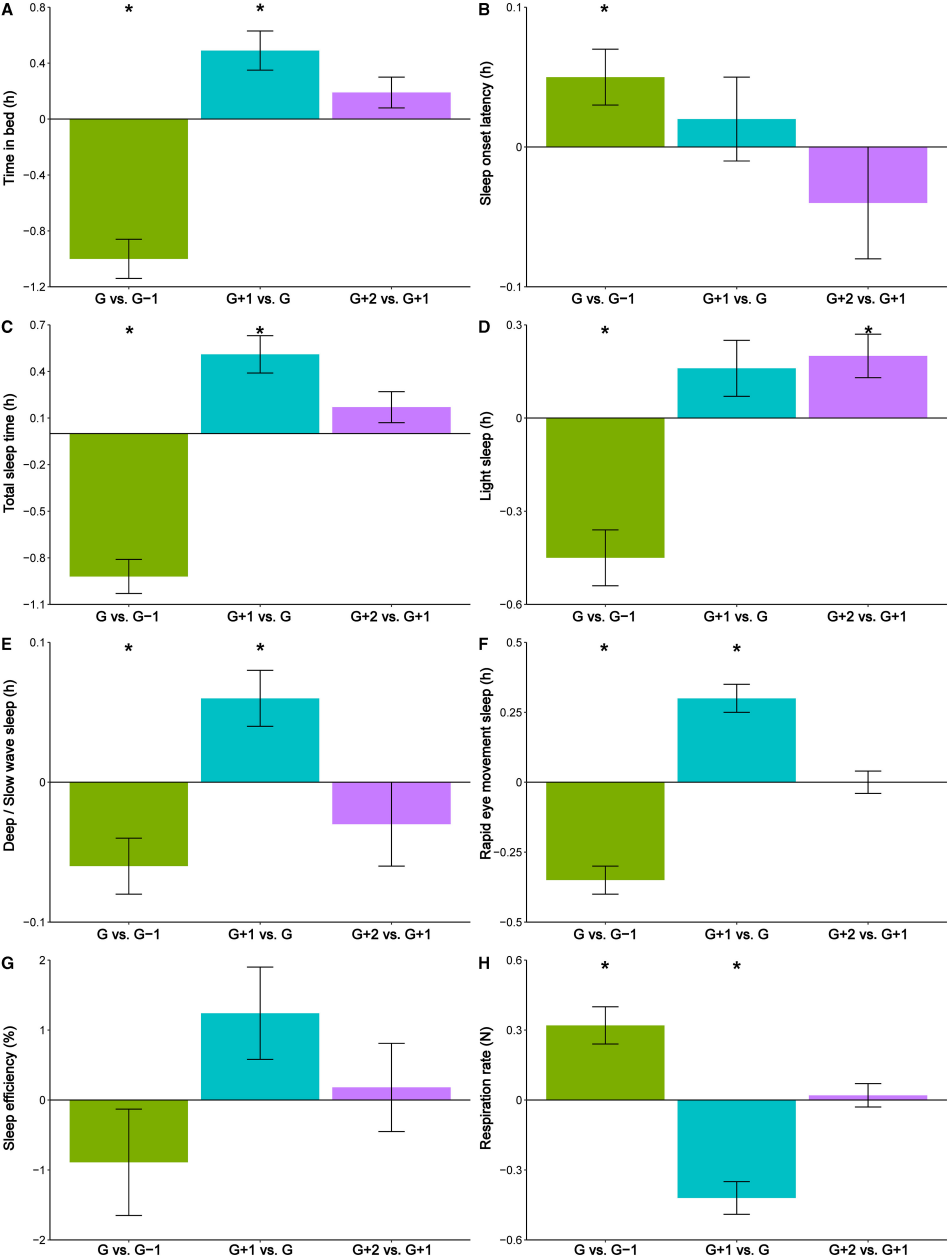
Visualization of the within-athlete variations in the measured sleep variables [time in bed **(A)**, sleep-onset latency **(B)**, total sleep time **(C)**, light sleep **(D)**, deep sleep **(E)**, rapid eye movement (REM) sleep **(F)**, sleep efficiency **(G)**, and respiration rate in non-REM sleep **(H)**] across the different soccer game days: game night vs. night before game (G vs. G-1, green bars), first night after the game vs. game night (G+1 vs. G, blue bars), and second night after the game vs. first night after the game (G+2 vs. G+1, purple bars). Data are based on sleep monitoring in 29 elite female soccer players. The error bars represent the SE; ^*^Represents a significant change, *p* < 0.05.

During game night (G), random intercept models showed that TIB decreased by 60.0 ± 8.4 min (95% CI [−76.46, −43.54], *p* = <0.001), TST decreased by 55.2 ± 6.6 min (95% CI [−68.14, −42.06], *p* = <0.001), LS decreased by 27.0 ± 5.4 min (95% CI [−37.58, −16.42], *p* = <0.001), SWS decreased by 3.6 ± 1.2 min (95% CI [−5.95, −1.25], *p* = 0.001), and REM decreased by 21.0 ± 3.0 min (95% CI [−26.88, −15.12], *p* = <0.001), compared to the night before the game (G-1). Further, G was associated with increases in SOL, by 3.0 ± 1.2 min (95% CI [0.65, 5.35], *p* = 0.013) and in NREM RPM, by 0.32 ± 0.08 respirations per minute (95% CI [0.16, 0.48], *p* = <0.001), compared to G-1. These variations are visually shown in Figure 2, green bars. G explained 0.5% of the variation in SOL, 0.9% of the variation in SWS, 5.9% of the variation in NREM RPM, 6.3% of the variation in LS, 11.9% of the variation in TIB, 12.9% of the variation in REM, and 15.0% of the variation in TST. The ICC value showed that 6–29% of the total variance in the sleep variables was due to differences between participants, while 86% of the total variance in NREM RPM was due to differences between participants. Between-athlete variances were significant for SOL (*p* < 0.001), TST (*p* = 0.026), SWS (*p* < 0.001), REM (*p* = 0.005), SE (*p* < 0.001), and NREM RPM (*p* < 0.001). Full results for the ICC values and between-athlete in sleep, comparing the effect of G to G-1, are presented in Table 2(A).

**Table 2:** The ICC values and between-athlete variance in sleep obtained from multilevel, random intercept regression analyses testing the effects of the analyzed game night periods (IV) on sleep (DV), based on data from 29 female elite football players.

	**(A) Effect of game night (IV, 0** **=** **G-1, 1** **=** **G) on sleep (DV)**					**(B) Effect of first day after the game (IV, 0** **=** **G, 1** **=** **G+1) on sleep on sleep (DV)**					**(C) Effect of second day after the game (IV, 0** **=** **G+1, 1** **=** **G+2) on sleep on sleep (DV)**				
**DV**	**ICC**	**Est**.	**S.E**.	**95% CIs**	**Sig**.	**ICC**	**Est**.	**S.E**.	**95% CIs**	**Sig**.	**ICC**	**Est**.	**S.E**.	**95% CIs**	**Sig**.
Time in bed (h)	0.10	0.22	0.14	−0.05, 0.49	0.118	0.12	0.26	0.10	0.06, 0.46	*0.011*	0.17	0.30	0.08	0.14, 0.46	*<0.001*
Sleep-onset latency (h)	0.11	0.02	0.00	0.01, 0.03	*<0.001*	0.10	0.02	0.01	<0.01, 0.04	*0.001*	0.12	0.02	0.01	<0.01, 0.04	*<0.001*
Total sleep time (h)	0.09	0.14	0.06	0.02, 0.26	*0.026*	0.09	0.15	0.07	0.01, 0.29	*0.021*	0.10	0.13	0.05	0.03, 0.23	*0.007*
Light sleep (h)	0.06	0.05	0.04	−0.03, 0.13	0.221	0.06	0.05	0.03	−0.01, 0.11	0.054	0.10	0.07	0.02	0.03, 0.11	*<0.001*
Deep/slow wave sleep (h)	0.29	0.05	0.01	0.03, 0.07	*<0.001*	0.30	0.04	0.01	0.02, 0.06	*0.001*	0.33	0.05	0.02	0.01, 0.09	*0.002*
REM sleep (h)	0.12	0.03	0.01	0.01, 0.05	*0.005*	0.08	0.02	0.01	<0.01, 0.04	*0.018*	0.09	0.02	0.01	<0.01, 0.04	*0.005*
Sleep efficiency (%)	0.20	13.66	2.94	7.90, 19.42	* <0.001*	0.24	14.25	3.46	7.47, 21.03	* <0.001*	0.18	9.79	3.02	3.87, 15.71	*0.001*
NREM RPM (N)	0.86	2.64	0.67	1.33, 3.95	* <0.001*	0.88	2.82	0.72	1.41, 4.23	* <0.001*	0.90	2.59	0.70	1.22, 3.96	* <0.001*

*Regressions were clustered on participant. Values are unstandardized. Significant results are italicized. DV, dependent variable; ICC, intraclass correlation; Est., estimate; S.E., standard error; 95% CIs, lower, upper 95% confidence intervals; Sig., significance; REM, rapid eye movement sleep; NREM RPM, non-REM respirations per minute*.

During the first night after the game (G+1), random intercept models showed that TIB increased by 29.4 ± 8.4 min (95% CI [12.94, 45.86], *p* = <0.001), TST increased by 30.6 ± 7.2 min (95% CI [16.45, 44.71], *p* = <0.001), SWS increased by 3.6 ± 1.2 min (95% CI [1.25, 5.95], *p* = 0.004), and REM increased by 18.0 ± 3.0 min (95% CI [12.12, 23.88], *p* = <0.001), compared to game night (G). Further, during G+1, NREM RPM decreased by 0.42 ± 0.07 respirations per min (95% CI [−0.56, −0.28], *p* = <0.001), compared to G. These variations are visually shown in Figure 2, blue bars. G+1 explained 0.1% of the variation in SOL, 0.8% of the variation in LS and SE, 1.1% of the variation in SWS, 3.0% of the variation in TIB, 4.5% of the variation in TST, 9.2% of the variation in REM, and 11.0% of the variation in NREM RPM. The ICC value showed that 6–30% of the total variance in the sleep variables was due to differences between participants, while 88% of the total variance in NREM RPM was due to differences between participants. Between-athlete variances were significant for TIB (*p* = 0.011), SOL (*p* = 0.001), TST (*p* = 0.021), SWS (*p* = 0.001), REM (*p* = 0.018), SE (*p* < 0.001), and NREM RPM (*p* < 0.001). Full results for the ICC values and between-athlete in sleep, comparing the effect of G+1 to G, are presented in Table 2(B).

During the second night after the game (G+2), random intercept models showed that LS increased by 18.0 ± 4.2 min (95% CI [9.77, 26.23], *p* = 0.005) compared to the first night after the game (G+1). These variations are visually shown in Figure 2, purple bars. G+2 explained 1.6% of the variation in LS. The ICC value showed that 9–33% of the total variance in the sleep variables was due to differences between participants, while 90% of the total variance in NREM RPM was due to differences between participants. Between-athlete variances were significant for all sleep variables (TIB: *p* < 0.001; SOL: *p* < 0.001; TST: *p* = 0.007; LS: *p* < 0.001; SWS: *p* = 0.002; REM: *p* = 0.005; SE: *p* = 0.001; NREM RPM: *p* < 0.001). Full results for the ICC values and between-athlete in sleep, comparing the effect of G+2 to G+1, are presented in Table 2(C).

## Discussion

The current study of sleep in female elite soccer players aimed to investigate the bidirectional associations between perceived fatigue and variations in sleep over one full competitive season and the associations between soccer games and the sleep of players. It was expected that increased perceived fatigue would be associated with increases in the sleep of the players and their NREM RPM (H1). Since a soccer game represents the highest load for a female soccer player, it was expected that sleep on game nights would be disrupted, that NREM RPM would increase, and that the sleep of the players would normalize the nights following game nights (H2). The results in the current study confirmed these hypotheses. Increased perceived fatigue was associated with increased TIB and SWS, increased REM sleep was associated with decreased perceived fatigue the next day, and increased NREM RPM was associated with increased perceived fatigue the next day. Further, the night of soccer games was associated with shorter TIB, TST, LS, SWS, and REM sleep, longer SOL and increased NREM RPM, compared to the night before the games. The first night after the soccer games was associated with longer TIB, TST, SWS, and REM, and lower NREM RPM, compared to game night. The second night after the game was associated with increased LS, compared to the first night after the game.

### Perceptions of Fatigue of Players Are Associated With Variations in Sleep

In the context of the current research, the main function of sleep is to ensure adequate recovery of female soccer players. We applied the principle that increased experience of fatigue should be associated with longer sleep, as fatigue raises the need for recovery (Brand et al., 2009, 2010; Whitworth-Turner et al., 2018). The current study found significant associations between increased perceived fatigue of the players and longer TIB and SWS. Interestingly, TST was not affected. It seems that SWS, uniquely of all sleep stages, varies based on the increasing fatigue. The association between SWS and recovery is documented in previous research, such that growth hormones are secreted in SWS. Research also indicates that SWS has an inhibitory effect on the secretion of cortisol (Weitzman et al., 1983), making SWS an important part of the physiological recovery process of the athletes (Walters, 2002; Akerstedt and Nilsson, 2003). Together, these findings indicate that on days with increased perceived fatigue, players extend their TIB, and SWS, uniquely of all sleep stages, increases to allow for physiological recovery.

Increases in REM sleep were associated with subsequently decreased perceived fatigue. It is possible that these associations are related to the functioning of the hypothalamic–pituitary–adrenal (HPA) axis, which modulates the stress response system by stimulating the adrenal release of cortisol (Chrousos et al., 2016). The HPA axis is activated in light of inflammatory processes in cells. In female soccer players, training sessions involve demanding physical efforts (e.g., high-intensity sprinting, accelerations, retardations, hard tackles), which may lead to muscle damage, followed by inflammatory processes in muscle cells (Nédélec et al., 2013). Previously, cortisol has been shown to reduce inflammation (Braun and Marks, 2015). Despite the lack of direct evidence in the present study, it may be postulated that REM sleep may have a restorative function in muscle cells, thereby reducing the perception of fatigue among female elite soccer players.

Contrary to the effect of REM, increases in NREM RPM were associated with subsequently increased perceived fatigue. Seeing as respiration rate may represent a physiological correlate of psychological well-being with autonomic reactivity (Sakakibara and Hayano, 1996; Van Diest et al., 2006), increased NREM RPM and its association with increased fatigue may signal psychological stress among the female soccer players. It is well-established that the competitive season represents a stressor for soccer players, a period also associated with poorer subjective recovery (Faude et al., 2011). The present findings may be used to inform future research, which will need to support the roles of REM sleep and NREM RPM in perceived fatigue with objective indices of recovery, psychological stress, and inflammatory responses.

### Soccer Games Disrupt Sleep on Game Night

Soccer players obtain shorter TIB and TST after playing soccer games, compared to the night before the games. This may be attributed to the cognitive and emotional activity induced by soccer games (Williams and Ford, 2013; Nédélec et al., 2015b; Williams and Jackson, 2019), which likely disrupts the ability of the players to fall asleep. Moreover, increased NREM RPM occurs on nights of soccer games. This indicates that the recovery processes of soccer players may be hampered, since raised RPM points to inflammatory processes in cells and/or raised immunological activity (Davies and Maconochie, 2009), and to disruptions in psychological well-being and autonomic reactivity (Sakakibara and Hayano, 1996; Van Diest et al., 2006). Increased SOL supports this line of thought, as higher SOL indicates sleep disturbances on nights of soccer games. This is an important finding for the field of practice, since subsequent training loads should be adapted to the disrupted sleep, in order to prevent possible negative effects of overtraining the day after a game. However, the causality between variations in sleep and the disrupting effects of game nights need to be tested in future research with appropriate experimental designs, utilizing objective indices of the inflammatory responses and psychological well-being of the players.

Sleep stages decreased on game night likely as a consequence of the observed decrease in TST. However, the roles of the different sleep stages in the recovery processes of players allow us to hypothesize about the consequences of the variations in LS, SWS, and REM. Physical and mental efforts of the players peak during soccer games (Furley and Memmert, 2013), which likely leads to an increased need for recovery (Smith et al., 2018; Whitworth-Turner et al., 2018). Physiological and muscular recovery during the night occurs in SWS (Dijk, 2009), which was reduced on game night in the current study. This indicates that players do not obtain an adequate recovery on game nights. The concurrent decrease in REM raises further questions about the recovery of the players on game nights. REM is important for the regulation of the emotions of the players (Goldstein and Walker, 2014). REM was likely higher the night before soccer games (G-1), compared to game night (G; see Figure 1). Soccer players may have experienced high emotional activity before soccer games, due to the insecurity and excitement associated with the outcomes of games, increasing their stress levels (Filaire et al., 2001; O'Donnell et al., 2018). Thus, the decreased REM following a soccer game might be attributed to the experience of relief of the players after finishing the soccer games (Nixdorf et al., 2015). Relief is an emotion that typically occurs after experiencing tension, insecurity, and anxiety. All such emotions are normal emotional reactions to competitions and soccer games, especially the important games elite international soccer players engage in. When players experience relief, they become more relaxed and comfortable, and thus, the need for regulation of emotions is reduced, thus possibly reducing REM. Future research should investigate the role of emotional activity that occurs the day before games, and REM sleep of the players. Overall, the reduced TIB, TST, time in sleep stages LS, SWS, and REM, and increased SOL and NREM RPM, indicate that the recovery of female soccer players may be disrupted on game nights.

### Sleep of the Players Normalizes on the First and Second Night After the Game

Female elite soccer players obtain longer TIB, TST, SWS, and REM, and lower NREM RPM during the first night after the game, compared to game night. Further, LS increases the second night after the game compared to the first night after the game. Overall, these results show that the sleep of female soccer players improves on the night after the game, indicating an increased, more optimal recovery. LS is upregulated on the second night after the game, showing a further adjustment toward normal, optimal sleep. Interestingly, previous research indicates that one night with disrupted sleep following a soccer game does not disrupt the subsequent physical explosive performance of players (e.g., countermovement jump height), nor the subjective well-being of the players, or cognitive function, assessed the following morning (Abbott et al., 2020). Therefore, the sleep disruption on game night, and the subsequent normalization of sleep on the first and second nights after the game likely do not induce disruptions to the mental and physical functioning and performance of the players.

### Limitations and Strengths

Several limitations should be kept in mind when interpreting the current results. First, the study could have benefitted from a bigger sample size. The low number of participants in the current study may have influenced the power to detect significant associations in the investigated multilevel statistical analyses. Second, relevant variables that may have influenced the sleep of the players were not considered in the present study, such as training load, ratings of perceived exertion, mental stress loads, time of game plays, as well as sleep during daytime and napping frequency. Third, 25.5% of all potential sleep data were lost mainly because of difficulties with the local wi-fi network, especially when the players stayed at different hotels outside their homes. Some players also forgot to bring the sleep monitor at some training camps or game days when the nights were spent outside their homes. However, the current study has several strengths as well. First, the participants in the current study are female international soccer players who are qualified for the Norwegian national team. Second, the current study is the first to examine the bidirectional associations between perceived fatigue and objectively quantified sleep including sleep staging, and the sleep variations during and after soccer games, across an entire competitive soccer period lasting ~4 months.

### Conclusion

Overall, the current study highlights the substantial individual sleep variability in association with perceived fatigue and game periods. Perceived fatigue has bidirectional associations with TIB, sleep stages (SWS and REM), and NREM RPM. The variations in fatigue of athletes and the implicated sleep stages, as well as TIB and respiration rate in NREM sleep, are relevant for the recovery of soccer players. Further, on game nights, female elite soccer players seem to obtain suboptimal recovery because of the extraordinary loads caused by soccer games. In the nights following soccer games, sleep of the players normalizes, reducing the likelihood of negative performance-related consequences linked to the disrupted sleep on game night.

### Implication and Further Research

Based on the results in the current study, we propose the implementation of an individual approach to monitor sleep and game loads, to better understand how players cope with training loads, and in turn, how these factors influence their recovery (Costa et al., 2019). Future studies should investigate the use of recovery strategies for improvement in sleep after high training loads, such as sleep hygiene, active recovery, and dietary strategies, as well as environmental factors such as light exposure (Nédélec et al., 2015b; Falkenberg et al., 2021). Future studies should also include GPS systems and HR monitoring to objectively detect workload. The complexity of recovery in soccer reinforces the need for future research to estimate the quantitative importance of the fatigue mechanisms and identify the potential factors influencing sleep in elite soccer players.

## Data Availability Statement

The raw data supporting the conclusions of this article will be made available by the authors, without undue reservation.

## Ethics Statement

The studies involving human participants were reviewed and approved by REC Central, the Regional Committee for Medical and Health Research Ethics (REC) in Central Norway, founded on the Norwegian law on research ethics and medical research, has approved the study (project ID 2017/2072/REK midt). The patients/participants provided their written informed consent to participate in this study.

## Author Contributions

FM, GH, and MH contributed to the conception and design of the study. FM and MH performed the statistical analysis. FM wrote the first draft of the manuscript. MO, FM, and MH organized the database. MO wrote sections of the manuscript. MH contributed to the main revision of the manuscript. All authors contributed to the final manuscript revision, read, and approved the submitted version.

## Conflict of Interest

The authors declare that the research was conducted in the absence of any commercial or financial relationships that could be construed as a potential conflict of interest.

## Publisher's Note

All claims expressed in this article are solely those of the authors and do not necessarily represent those of their affiliated organizations, or those of the publisher, the editors and the reviewers. Any product that may be evaluated in this article, or claim that may be made by its manufacturer, is not guaranteed or endorsed by the publisher.

## Appendix

**Table A1:** Multilevel, random intercept regressions investigating the effect of perceived fatigue (IV) on sleep variables (DVs), based on perceived fatigue and sleep data in 29 female football players.

	**Within-level effect of perceived fatigue on sleep**					**Between-level variance in sleep**			
**DV**	**ICC**	**Est**.	**S.E**.	**95% CIs**	**Sig**.	***R*** ^**2**^ **(%)**	**Est**.	**S.E**.	**95% CIs**	**Sig**.
Time in bed (h)	0.07	0.06	0.03	<0.01, 0.12	*0.037*	0.4	0.17	0.05	0.07, 0.27	0.001
Sleep-onset latency (h)	0.15	0.01	0.01	−0.01, 0.03	0.235	0.2	0.04	0.01	0.02, 0.06	<0.001
Total sleep time (h)	0.07	0.05	0.02	0.01, 0.09	0.052	0.3	0.13	0.04	0.05, 0.21	0.003
Light sleep (h)	0.05	0.02	0.02	−0.02, 0.06	0.258	0.1	0.05	0.02	0.01, 0.09	0.032
Deep sleep (h)	0.27	0.02	0.01	<0.01, 0.04	*0.007*	0.5	0.04	0.01	0.02, 0.06	<0.001
REM sleep (h)	0.07	0.01	0.01	−0.01, 0.03	0.102	0.2	0.02	0.01	<0.01, 0.04	<0.001
Sleep efficiency (%)	0.11	0.07	0.15	−2.22, 0.36	0.645	<0.1	9.05	2.35	4.44, 13.66	<0.001
NREM RPM (N)	0.89	0.01	0.01	−0.01, 0.03	0.337	0.1	2.38	0.61	1.18, 3.58	<0.001

*DV, dependent variable; ICC, intraclass correlation ; Est., estimate; S.E., standard error; 95% CIs, lower, upper 95% confidence intervals; Sig., significance; R^2^, within-athlete explained variance; REM, rapid eye movement; NREM RPM, non-REM respirations per minute. Regressions were clustered on participant. Values are unstandardized. Significant results are italicized*.

**Table A2:** Multilevel, random intercept regressions investigating the effect of sleep variables (IVs) on the next-day perceived fatigue (DV) on based on perceived fatigue and sleep data in 29 female football players.

	**Within-level effect of sleep on the next-day perceived fatigue**					**Between-level variance in the next-day perceived fatigue**				
**IVs**	**ICC**	**Est**.	**S.E**.	**95% CIs**	**Sig**.	***R*^**2**^ (%)**	**Est**.	**S.E**.	**95% CIs**	**Sig**.
Time in bed (h)	0.28	−0.02	0.03	−0.08, 0.04	0.511	<0.1	0.82	0.26	0.31, 1.33	0.002
Sleep-onset latency (h)	0.28	0.17	0.12	−0.07, 0.41	0.156	0.2	0.81	0.25	0.32, 1.30	0.001
Total sleep time (h)	0.28	−0.04	0.04	−0.12, 0.04	0.222	0.1	0.82	0.26	0.31, 1.33	0.002
Light sleep (h)	0.28	0.00	0.05	−0.07, 0.13	0.949	<0.1	0.82	0.27	0.29, 1.35	0.002
Deep sleep (h)	0.28	−0.09	0.10	−0.29, 0.11	0.386	0.1	0.82	0.26	0.31, 1.33	0.002
REM sleep (h)	0.28	−0.21	0.08	−0.37, −0.05	*0.008*	0.5	0.82	0.26	0.31, 1.33	0.002
Sleep efficiency (%)	0.28	−0.01	0.01	−0.03, 0.01	0.294	0.1	0.82	0.26	0.31, 1.33	0.002
NREM RPM (N)	0.27	0.27	0.09	0.09, 0.45	*0.002*	7.2	0.82	0.19	0.45, 1.19	<0.001

*DV, dependent variable; ICC, intraclass correlation; Est., estimate; S.E., standard error; 95% CIs, lower, upper 95% confidence intervals; Sig., significance; R^2^, within-athlete explained variance; REM, rapid eye movement; NREM RPM, non-REM respirations per minute. Regressions were clustered on participant. Values are unstandardized. Significant results are italicized*.
